# Bone Marrow Harbors a Unique Population of Dendritic Cells with the Potential to Boost Neutrophil Formation upon Exposure to Fungal Antigen

**DOI:** 10.3390/cells11010055

**Published:** 2021-12-24

**Authors:** Marieke Goedhart, Edith Slot, Maria F. Pascutti, Sulima Geerman, Timo Rademakers, Benjamin Nota, Stephan Huveneers, Jaap D. van Buul, Katherine C. MacNamara, Carlijn Voermans, Martijn A. Nolte

**Affiliations:** 1 Department of Hematopoiesis, Sanquin Research, Plesmanlaan 125, 1066 CX Amsterdam, The Netherlands; m.goedhart@hezelburcht.com (M.G.); edith.sloat@sanquin.nl (E.S.); m.f.pascutti@lumc.nl (M.F.P.); sulima_geerman@hotmail.com (S.G.); c.voermans@sanquin.nl (C.V.); 2 Molecular Cell Biology Lab, Department of Molecular Hematology, Sanquin Research, Plesmanlaan 125, 1066 CX Amsterdam, The Netherlands; t.rademakers@maastrichtuniversity.nl (T.R.); s.huveneers@amsterdamumc.nl (S.H.); j.vanbuul@sanquin.nl (J.D.v.B.); 3 Department of Molecular Hematology, Sanquin Research, Plesmanlaan 125, 1066 CX Amsterdam, The Netherlands; benjamin.nota@gmail.com; 4 Landsteiner Laboratory, Academic Medical Centre, University of Amsterdam, 1105 AZ Amsterdam, The Netherlands; 5 Department of Immunology and Microbial Disease, Albany Medical College, Albany, NY 12208, USA; macnamk@amc.edu

**Keywords:** dendritic cells, bone marrow, hematopoiesis, granulopoiesis, fungal infection, zymosan, dectin-1

## Abstract

Apart from controlling hematopoiesis, the bone marrow (BM) also serves as a secondary lymphoid organ, as it can induce naïve T cell priming by resident dendritic cells (DC). When analyzing DCs in murine BM, we uncovered that they are localized around sinusoids, can (cross)-present antigens, become activated upon intravenous LPS-injection, and for the most part belong to the cDC2 subtype which is associated with Th2/Th17 immunity. Gene-expression profiling revealed that BM-resident DCs are enriched for several c-type lectins, including Dectin-1, which can bind beta-glucans expressed on fungi and yeast. Indeed, DCs in BM were much more efficient in phagocytosis of both yeast-derived zymosan-particles and *Aspergillus conidiae* than their splenic counterparts, which was highly dependent on Dectin-1. DCs in human BM could also phagocytose zymosan, which was dependent on β1-integrins. Moreover, zymosan-stimulated BM-resident DCs enhanced the differentiation of hematopoietic stem and progenitor cells towards neutrophils, while also boosting the maintenance of these progenitors. Our findings signify an important role for BM DCs as translators between infection and hematopoiesis, particularly in anti-fungal immunity. The ability of BM-resident DCs to boost neutrophil formation is relevant from a clinical perspective and contributes to our understanding of the increased susceptibility for fungal infections following BM damage.

## 1. Introduction

The bone marrow (BM) is the primary organ responsible for the formation of all blood cell lineages, and also serves as a reservoir for immunological memory [1,2]. In addition, it harbors a small population of resident dendritic cells (DC) that can be primed by blood borne antigens and subsequently activate naïve T cells [3,4]. Thus, the BM can also act as a secondary lymphoid organ with capability of initiating adaptive immune responses.

DCs are professional antigen presenting cells and are key integrators of immune signals. Depending on environmental factors, such as PAMPs and inflammatory cytokines from innate immune cells, DCs produce cytokines and express cell surface molecules to skew newly primed T cells to the appropriate response. In the BM, DCs are located around the vasculature [3,5], the same location that has been described as the perivascular niche for hematopoietic stem cells (HSCs) [6,7]. However, hematopoiesis is rather sensitive to inflammation. On the one hand, inflammatory cytokines that are produced during an infection can instruct hematopoietic stem and progenitor cells (HSPCs) to produce specific offspring that is able to fight the invading pathogen [8,9,10]. On the other hand, prolonged exposure of hematopoiesis to an inflammatory milieu results in anemia and eventual hematopoietic exhaustion [11,12]. Thus, unlike in other secondary lymphoid organs, a tight regulation of immune activation is required in the BM. We therefore questioned whether BM-resident DCs are different from spleen-resident DCs regarding phenotype, function, and inflammatory profile. We discovered that, in fact, BM DCs are fully capable of activating T cells and responding to systemic infection with an inflammatory profile. BM DCs express high levels of c-type lectin receptors and are particularly capable of taking up fungal antigens in a Dectin-1 dependent manner. In addition, we showed that upon exposure to fungal antigens, BM DCs skew hematopoiesis towards increased neutrophil formation in a G-CSF dependent manner. Our data therefore reveal a role for BM DCs as translators between infection and hematopoiesis, particularly in anti-fungal immunity.

## 2. Materials and Methods

### 2.1. Mice and Intravenous Injections

C57BL/6J mice and C57BL/6J.OT-I TCR (OT-I) transgenic mice were maintained in the animal facility of the Netherlands Cancer Institute (Amsterdam, The Netherlands) under specific pathogen-free conditions. Mice were generally 8–14 weeks old at the start of the experiment. All animal experiments were approved by the Experimental Animal Committee of the Netherlands Cancer Institute, according to institutional and national guidelines.

In some experiments, mice were intravenously injected with 5 µg ultra-pure lipopolysaccharide (LPS) from E. coli K12 (Invivogen, Toulouse, France), 3 µg biotin-labelled CD11c antibody (clone N418; eBioscience, now Thermo Fisher, Waltham, MA, USA), or 100 µg FITC-labelled zymosan. FITC-labelled zymosan was prepared as follows: unlabeled zymosan (Sigma, Burlington, MA, USA) was resuspended in PBS at 25 mg/mL and heated for 30 min at 100 °C. This suspension was pelleted by centrifugation and washed twice with sterile PBS. Subsequently, the zymosan particles were resuspended at 5 mg/mL in PBS and incubated with 2.5 µg/mL fluoroscein-isothiocyanate (FITC; Sigma, Burlington, MA, USA) for 45 min at RT. Finally, zymosan particles were washed 5 times in sterile PBS, resuspended to a concentration of 5 mg/mL and stored at –20 °C.

### 2.2. Murine Cell Isolation

Murine BM cells were obtained by mildly crushing femurs and tibiae, and in some cases pelvi, humeri, and sternum, with a mortar and pestle. BM cell suspensions were filtered through a 70-μm cell strainer. Single splenocyte suspensions were prepared by crushing the spleen through a 70-μm cell strainer with the plunger of a syringe.

### 2.3. Human BM Cell Isolation

BM samples were obtained from patients (age range 58–80 years) undergoing median sternotomy for cardiac surgery, after informed consent and approval of the medical ethical review board of the AMC (MEC:04/042#04.17.370). BM mononuclear cells were isolated by density gradient centrifugation (Ficoll-paque PLUS, GE Healthcare, Freiburg, Germany).

### 2.4. Flow Cytometry, Cell Sorting and ImageStream

For flow cytometric analysis, cell sorting, and ImageStream analysis of murine DCs, BM cells were stained with antibodies against CD11c (clone N418) and MHCII (clone M5/114.15.2), and in some experiments for CD11b (clone M1/70), CD8 (clone 53-6.7), and CD24 (clone M1/69). For cell sorting, mononuclear cells were first enriched for CD11c^+^ cells with CD11c microbeads (Miltenyi Biotec, Leiden, The Netherlands). The following antibodies were used for phenotypic analysis of murine DCs: CD40 (clone 1C10), CD80 (clone 16-10A1), CD86 (clone GL1), and Dectin-1 (clone bg1fpj). For cell sorting of CD8b^+^CD44^−^ naïve CD8 T-cells from OT-I mice, OT-I splenocytes were enriched for CD8 T-cells with CD8 microbeads (Miltenyi Biotec) and cells were stained with antibodies against CD8b (clone H35-17.2) and CD44 (clone IM7). For cell sorting of murine LSK cells, BM cells were stained for biotin-labelled lineage markers (CD4 (clone GK1.5), CD8 (clone 53-6.7), B220 (clone RA3-6B2), CD11b (clone M1/70), Gr-1 (clone RB6-8C5, which recognizes mostly Ly6G and to some extent also Ly6C) and Ter119 (clone TER-119)), Sca-1 (clone D7), c-kit (clone 2B8), and enriched for progenitors by negative depletion of lineage cells using streptavidin microbeads (Miltenyi Biotec). Lineage-depleted BM was stained for Sca-1 (clone D7) and c-kit (clone 2B8). For analysis of granulopoiesis in zymosan-injected mice, BM cells were stained with antibodies against CD4 (clone GK1.5), CD8 (clone 53-6.7), B220 (clone RA3-6B2), Ter119 (clone TER-119), c-kit (clone 2B8), CD34 (clone RAM34), and Ly6G (clone clone 1A8). All antibodies were obtained from eBioscience, unless indicated otherwise. FACSCanto II (BD Biosciences, Franklin Lakes, NJ, USA) and FACSAria (BD Biosciences) were used for flow cytometric analysis and cell sorting. ImageStream Mark II Imaging Flow Cytometer (Merck Millipore, Burlington, MA, USA) was used for ImageStream analysis.

### 2.5. RNA Sequencing

For sorting, CD11c^+^ cells were enriched with CD11c microbeads (Miltenyi Biotec) from pooled BM and spleen (2 mice/sort, 3 sorts in total), and CD11c^+^MHCII^+^CD11b^high^CD24^low^ cDC2 were sorted. RNA was extracted with RNeasy Micro kit (Qiagen) according to the manufacturers protocol. RNA quality check, polyA RNA selection, and sequencing were performed at GenomeScan BV (Leiden, The Netherlands). RNA quality and quantity was determined by a Bioanalyzer Picochip (Agilent). Per sample, 25 ng RNA was used for library preparation and single-end, 75 nucleotide fragments were analyzed by Illumina NextSeq 500. Reads were aligned to the mouse genome (mm10) using STAR (v2.5.3a). Reads that aligned to (exonic parts of) genes annotated by Ensembl (release 81) were quantified using featureCounts (v1.5.2). Low abundant genes were removed for further analysis, genes with more than 1 CPM (counts per million) in at least 3 samples were kept. Differentially expressed genes between BM cDC2s and spleen cDC2s were assessed with limma trend, using a paired design. Genes with >1.5 fold change difference and FDR-adjusted *p*-values < 0.05 were considered significantly differentially expressed. Pair-wise Pearson’s correlation coefficients between the samples were calculated based on log_2_ CPM values, and grouped by hierarchical clustering. Gene ontology (GO) enrichment analysis with goseq (v1.30.0) was done for the significantly differentially expressed genes, using only GO-terms with curated evidence codes, meaning that GO-terms with electronic annotation evidence code (IEA) were removed. Significantly enriched GO-terms (adjusted *p*-value < 0.05) were clustered with a customized a gogadget (v2.1) script. Data are accessible through GEO Series accession number GSE182984 (https://www.ncbi.nlm.nih.gov/geo/query/acc.cgi?acc=GSE182984, accessed on 31 August 2021).

### 2.6. Confocal Microscopy

Murine bones were mounted directly in Tissue-Tek O.C.T. embedding compound (Sakura Finetek, Alphen aan den Rijn, The Netherlands) and slowly frozen while floating on liquid nitrogen. Then, 8 μm thick cryosections were prepared using the CryoJane^®^ Tape-Transfer System (Leica Biosystems, Amsterdam, The Netherlands). Sections were air-dried, fixed for 10 min in dehydrated acetone, and blocked with 5% BSA/PBS. Antibodies used for immunolabelling of BM sections were CD11c (clone N418; eBioscience), MHCII (clone M5/114.15.2; eBioscience), CD144 (VE-cadherin; clone BV13; Biolegend), and CD31 (PECAM; clone MEC 13.3; BD Biosciences). All sections were counterstained with Hoechst 33342 (Thermo Fisher, Waltham, MA, USA) and mounted with Mowiol. Images were acquired using a Leica TCS SP8 confocal microscope in conjunction with Leica Application Suite X software.

### 2.7. CD8 T-Cell Stimulation

BM DCs were loaded with OVA_257–264_ peptide (1nM; Invivogen) or Ovalbumin (100 ug/mL; Biosearch Technologies, Hoddesdon, UK). CD8b^+^CD44^−^ naïve OT-I T cells were sorted from OT-I splenocytes and labelled with CellTrace Violet (ThermoFisher Scientific) according to the manufacturer’s protocol. BM DCs were co-cultured with CD8 T cells for 3 days in 96-well plates with 25,000 DCs and 50,000 T cells per well in IMDM medium (ThermoFisher Scientific) supplemented with 10% FCS and 1% penicillin-streptomycin. Cells were cultured at 37 °C in a humidified incubator at 5% CO_2_. After 3 days, proliferation of CD8 T cells was determined by flow cytometry.

### 2.8. DC Co-Cultures with Zymosan and Aspergillus

For murine zymosan-uptake studies, CD11c^+^ enriched cells from BM and spleen were co-cultured for 2 h with 10–40 ug/mL FITC-labeled zymosan in RPMI-1640 (ThermoFisher Scientific) supplemented with 10% FCS and 1% penicillin-streptomycin. In some experiments, 20 µg/mL Dectin-1 blocking antibody (clone R1-8g7; Invivogen; [13,14,15,16]) was added to the culture. For human zymosan-uptake studies, human BM mononuclear cells were enriched for DCs with CD11c microbeads (Miltenyi Biotec, Leiden, The Netherlands) and co-cultured with 40 µg/mL zymosan for 2 or 16 h in RPMI-1640 (ThermoFisher Scientific, Waltham, MA, USA) supplemented with 10% FCS and 1% penicillin-streptomycin in the presence of blocking antibodies against CD11b (10 ug/mL; clone 44A; American Type Culture Collection) and CD18 (10 ug/mL; clone IB4; American Type Culture Collection) [17,18]. For *Aspergillus*-uptake studies, conidia from *A. fumigatus* strain AfS35 (D. Hartl, Tuebingen, Germany) (3 × 10^6^/mL) were incubated with 250 µg/mL FITC (Sigma) in PBS for 30 min at 37 °C, washed 3 times with PBS and used directly. Murine DCs were cultured in a 1:1 ratio with FITC-labelled *Aspergillus conidia* in RPMI-1640 (ThermoFisher Scientific) supplemented with 10% FCS and 1% penicillin-streptomycin for 16 h in the presence of 20 µg/mL Dectin-1 blocking antibody (clone R1-8g7; Invivogen, Toulouse, France). Uptake of zymosan and Aspergillus by DCs was determined by flow cytometry. To produce supernatant from murine BM DCs for LSK cultures, sorted CD11c^+^MHCII^+^ BM DCs were co-cultured for 16 h with 40 µg/mL zymosan (Sigma) in X-VIVO 15 medium (Lonza, Basel, Switzerland). All cultures were performed at 37 °C in a humidified incubator at 5% CO_2_.

### 2.9. LSK Cell Culture Assays

For 11-day LSK cultures, LSK cells (Lin^−^c-Kit^−^ Sca-1^+^) were sorted to purity and cultured in 96-well plates with 4000 cells per well in X-VIVO 15 medium (Lonza) with 2 ng/mL thrombopoietin (TPO) and stem cell factor (SCF) and 5 ng/mL Flt3-L. LSK cultures contained 25% supernatant from 16 h co-cultures of BM DCs with and without zymosan in X-VIVO 15 medium. In some experiments, 5 µg/mL G-CSF blocking antibody (clone 67604; R&D Systems, Minneapolis, MN, USA) or Rat IgG1 isotype control (Biolegend, San Diego, CA, USA) was added. Cells were cultured at 37 °C in a humidified incubator at 5% CO_2_. All cytokines were obtained from Peprotech (London, UK).

### 2.10. Statistics

Mean values plus or minus standard deviation (SD) or standard error of the mean (SEM) are shown. Two-way ANOVA and two-tailed unpaired *t*-tests, paired *t*-tests, and multiple *t*-tests were performed as indicated to determine statistical significance. *p* < 0.05; ** *p* < 0.01; *** *p* < 0.001.

## 3. Results

### 3.1. BM Harbors a Substantial Population of Dendritic Cells

**Figure 1:**
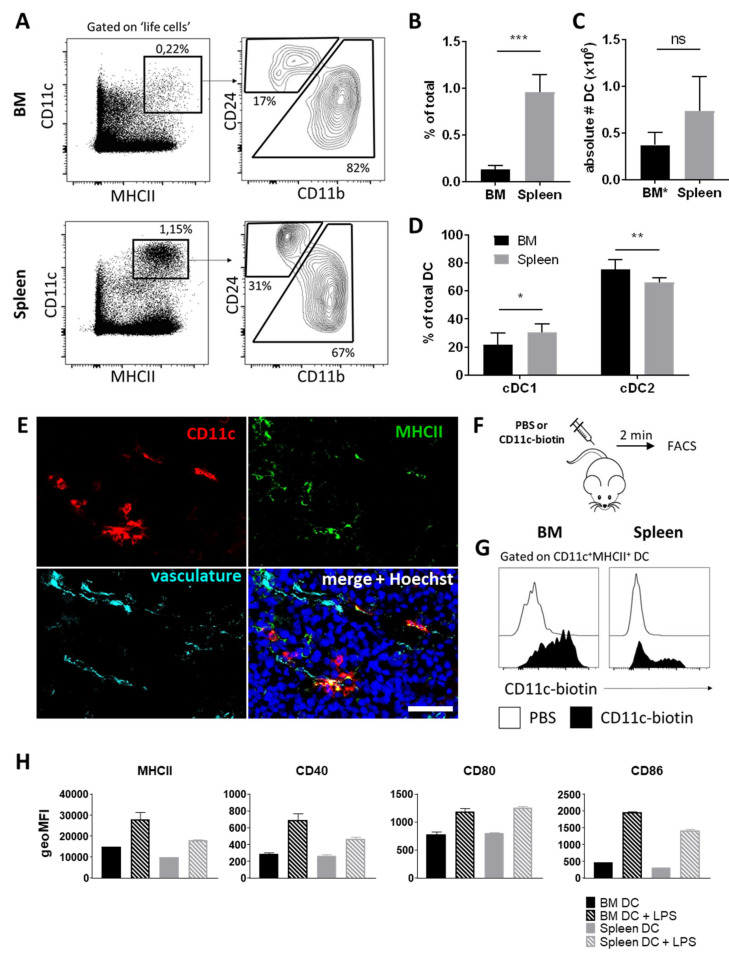
BM harbors a substantial population of DCs with an inflammatory profile. (**A**) Representative FACS plots of DCs and DC subsets in BM and spleen. (**B**,**D**) Percentages and (**C**) total numbers of DCs and DC subsets in BM and spleen. (Mean ± SD, *n* = 5–7, paired *t*-tests). *Total number of DCs in all BM containing bones were calculated per mouse by multiplying total number of BM DC derived from 2 femurs and 2 tibiae with the factor 3. (**E**) Cryosections of murine femurs stained for CD11c (red), MHCII (green), VE-cadherin and CD31 (cyan) and counterstained with Hoechst to visualize nuclei (blue). Original magnification = 630x. Scale bar = 50 µm. Representative image of 3 mice. (**F**) Experimental set-up for (**G**) in vivo binding of intravenously injected biotinylated CD11c to BM and splenic DCs within a 2-min time period. FACS plots are representative of 3 mice. (**H**) Quantification of geoMFI for expression of MHCII, CD40, CD80, and CD86 by DC from BM and spleen, in steady state, and 6 h after intravenous administration of LPS. (Mean ± SD, *n* = 2). * *p* < 0.05; ** *p* < 0.01; *** *p* < 0.001.

First, we set out to determine the frequency and localization of DCs in the BM as compared to the spleen. Flow cytometric analysis revealed that CD11c^+^MHCII^+^ DCs were relatively rare in the BM, ranging from 0.11 to 0.22% of life nucleated cells, which is significantly less than in the spleen (Figure 1A,B). We then calculated the total number of DCs present in all BM containing bones by multiplying total number of BM DCs derived from two femurs and two tibiae with the factor 3, as femurs and tibiae together contain one third of the BM in the murine body [19]. The total number of BM DCs did not significantly differ from the total number of splenic DCs (Figure 1C), which is related to the fact that the total volume of BM throughout the body is substantial, containing a vast number of total nucleated cells [19]. Of note, BM DCs typically expressed higher levels of MHCII than splenic DCs, which has been reported previously and possibly reflects a more activated state [5]. 

Conventional CD11c^+^MHCII^+^ DCs can be divided in two major subtypes. Type 1 DCs (cDC1) are important for anti-viral immunity, require the transcription factor IRF8, and are characterized by expression of CD24 or CD8 [20,21]. Type 2 DCs (cDC2) on the other hand are characterized by the expression of IRF4 and CD11b, and are important for Th2 and Th17 immunity [20,22,23]. Even though BM DCs typically expressed higher levels of CD11b than splenic DCs, as reported previously [5], we could discern cDC1 and cDC2 subsets based on CD11b and CD24 expression in both spleen and BM (Figure 1A). The majority of both spleen and BM DCs were of the cDC2, Th2/Th17 inducing variety: they constituted ~65% of total DCs in the spleen and were even more abundant in the BM at ~80% of total DCs (Figure 1D). Accordingly, CD8^+^ CD11b^−^ DC constituted ~20% of total splenic DCs and only ~5% of total BM DCs (Figure S1A,B).

### 3.2. Perivascular BM DCs Are Accessible to Blood-Borne Molecules and Respond to Systemic Infection

Previous work indicated that CX_3_CR1^+^ DCs form perivascular clusters in the BM [3,5], which suggests that they are accessible to blood borne molecules and have the capability to respond to systemic infection. We determined the localization of BM DCs on BM cryosections by staining for CD11c and MHCII, similar to the FACS-based characterization. We could indeed find BM DCs that are located close to the BM vasculature (Figure 1E), although we only observed single BM DCs and did not find any evidence for perivascular clusters of DCs. To determine whether BM DCs are readily accessible to blood-born molecules, we injected mice with a biotinylated CD11c antibody and sacrificed them after 2 min (Figure 1F). We used directly labelled antibodies against CD11c and MHCII to identify DCs with flow cytometry, and determined binding of the biotinylated CD11c antibody with streptavidin staining (Figure S1D). We found that splenic DCs were divided into a population that had bound the biotinylated CD11c antibody, most likely DCs that are located in the highly vascularized red pulp, and DCs that did not bind the injected antibody, due to their presence in the white pulp, which secludes them from blood stream (Figure 1G and Figure S1D). In contrast, all BM DCs had bound the biotinylated CD11c antibody within the 2-min time window, indicating that the entire BM DC population is readily accessible to blood-born molecules (Figure 1G and Figure S1D).

Next, we investigated whether BM DCs respond to intravenously injected lipopolysaccharide (LPS), a model for systemic infection. We predicted the response of BM DC to LPS to be less strong compared to splenic DCs, as a dampened inflammatory response could be beneficial to the bone marrows hematopoietic process and its precious HSCs. However, we in fact found that BM DCs showed equal or even higher upregulation of MHCII and the co-stimulatory molecules CD40, CD80, and CD86 in response to LPS compared to their splenic counterparts (Figure 1H). Thus, BM DCs showed an inflammatory profile in response to in vivo LPS challenge.

A hallmark that functionally separates DCs from other antigen presenting cells is their ability to activate naïve T cells. We could demonstrate that the CD11c^+^MHCII^+^ cells in the BM are indeed bona fide DCs, as they are capable of inducing proliferation of naïve OT-I CD8 T cell responses when loaded with OVA peptide antigens (Figure S2). A similar profile was observed when we used OVA protein, which indicates that BM DCs can also cross-present antigens and thereby activate naïve CD8 T cells to exogenous antigens (Figure S2). Together, these data indicate that the BM hosts a unique population of bona fide DCs that show a pro-inflammatory response to systemically applied stimuli to initiate protective immunity.

### 3.3. mRNA Profiling Reveals High Expression of C-Type Lectin Family Receptors by BM DCs

**Figure 2:**
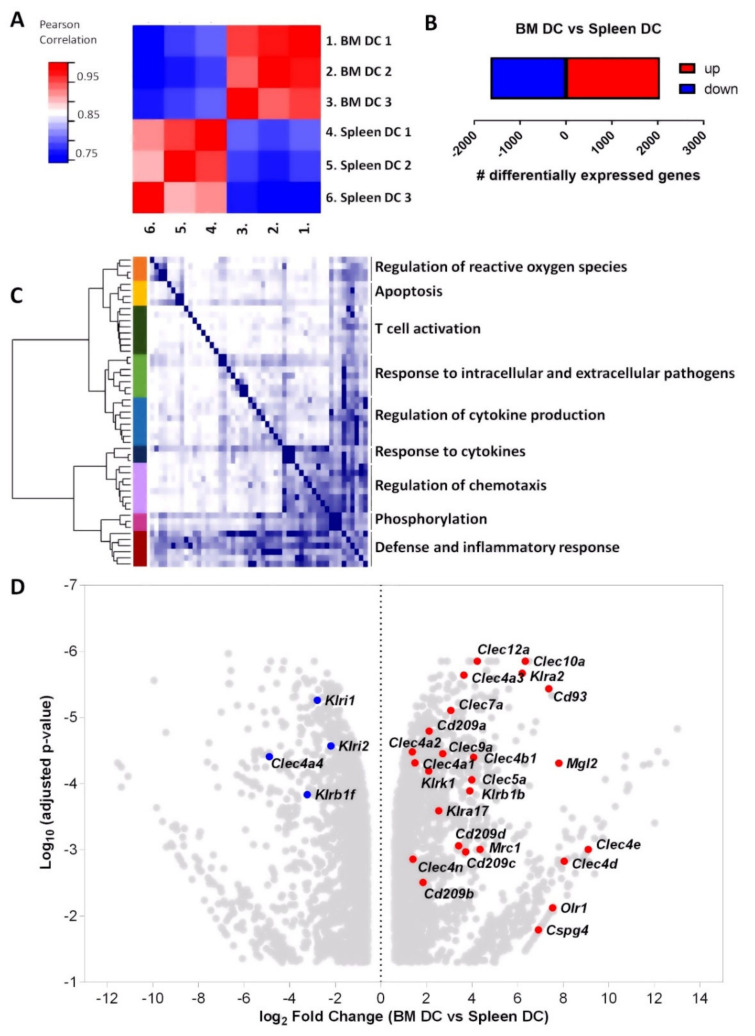
mRNA profiling reveals high expression of c-type lectin family receptors by BM DCs. RNAseq analysis was performed on cDC2 derived from BM and spleen. (**A**) Hierarchical clustering of the samples based on Pearson’s correlation coefficient. (**B**) Bar plot showing a total of 3697 differentially expressed (DE) genes being significantly up- (red, 2052) or downregulated (blue, 1645) in BM cDC2 as compared to splenic cDC2. (**C**) Hierarchical clustering of gene ontology (GO)-terms enriched in the DE expressed genes. In total 51 GO-terms were found enriched, which we clustered into 8 groups by comparison of their overlap index. Results are shown as a heatmap of the overlap indices, with the clustering and a color representation of each group on the left and a short description of the group on the right. The full heatmap is shown in Supplementary Figure S2. (**D**) Volcano plot of significantly up- and downregulated genes. Colored dots indicate genes encoding CTL/CTLD superfamily receptors being significantly up- (red) and downregulated (blue) in BM cDC2 as compared to splenic cDC2.

To investigate the differences between BM DCs and splenic DCs in further detail, we compared their transcriptional profiles. In order to make a fair comparison and exclude any differences due to the different distribution of the cDC1s and cDC2s BM and spleen, we sorted cDC2s from both organs and analyzed their transcriptome with RNA sequencing (RNAseq). Hierarchical clustering based on Pearson’s correlation coefficient revealed that BM DCs from three individual samples of pooled mice clustered together, and apart from splenic DCs (Figure 2A). In total, 3697 genes were differentially expressed more than 1.5-fold in BM DCs compared to splenic DCs (Figure 2B and Table S1). Gene ontology analysis revealed that many of the differentially expressed genes between BM DCs and splenic DCs were associated with inflammatory processes such as T cell activation, production of and response to cytokines, and defense- and inflammatory responses. (Figure 2C and Figure S3). Particularly, many receptors of the C-type lectin/C-type lectin-like domain (CTL/CTLD) superfamily were expressed higher in BM DCs than in splenic DCs (Figure 2D), which includes many pathogen-recognition receptors for bacterial and fungal cell surfaces [24,25]. Of the 29 CTL/CTLD superfamily receptors that were differentially expressed between BM DCs and splenic DCs, 25 had significantly higher expression levels in BM DCs (Figure 2D). Thus, BM DCs have an altered inflammatory transcriptional profile as compared to splenic DCs, with particularly high expression of many CTL/CTLD superfamily receptors, possibly suggesting a distinct role for BM DCs in the immune response against bacteria and/or fungi. 

### 3.4. BM DCs Express High Levels of Dectin-1, Which Mediate Uptake of Fungal Antigens

**Figure 3:**
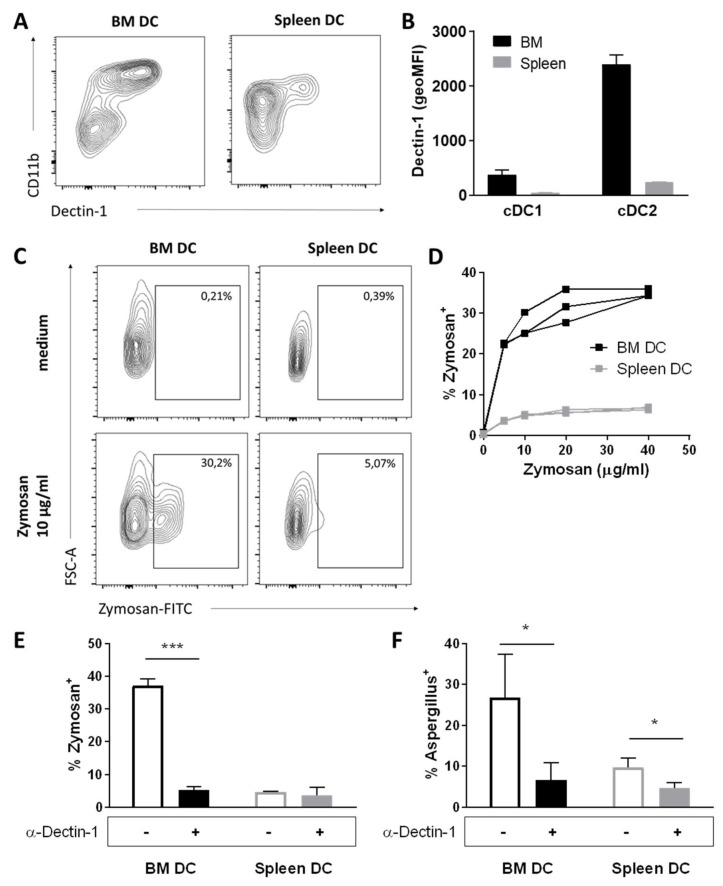
BM DCs express high, functional protein levels of the c-type lectin receptor Dectin-1. (**A**) Representative FACS plots and (**B**) quantification of geoMFI for expression of Dectin-1 by subsets in BM and spleen, as determined by flow cytometry (Mean ± SD, *n* = 2). (**C**) Representative FACS plots and (**D**) quantification of zymosan uptake by BM DCs and splenic DCs after 2 h in vitro co-culture (*n* = 3, *p* < 0.0001, 2-way ANOVA). (**E**) Uptake of zymosan particles by BM DCs and splenic DCs after 2 h in vitro co-culture in the presence or absence of a Dectin-1 blocking antibody (Mean ± SD, *n* = 3, multiple *t*-tests). (**F**) Uptake of life *Aspergillus conidia* by BM DCs and splenic DCs after overnight in vitro co-culture in the presence or absence of a Dectin-1 blocking antibody (Mean ± SD, *n*=3, multiple *t*-tests). * *p* < 0.05; *** *p* < 0.001.

One well known C-type lectin is CLEC7A, or DC-associated C-type lectin (Dectin-1), which recognizes β-glucan, a polysaccharide found on bacterial and fungal cell walls, and is a pivotal pathogen-recognition-receptor (PRR) in murine antifungal immunity [26]. The *Clec7α* gene was significantly expressed > 8 fold (log_2_ fold change = 3.068) higher in BM DCs compared to splenic DCs as determined by RNAseq (Figure 2D). We subsequently investigated protein expression of Dectin-1 by DCs with flow cytometry, and found that Dectin-1 was highly expressed by BM cDC2s, whereas their splenic counterparts expressed relatively low levels of Dectin-1 (Figure 3A,B), as reported previously [27]. The cDC1 in both BM and spleen had negligible Dectin-1 expression (Figure 3A,B).

To investigate whether the high expression of Dectin-1 by BM DCs made them particularly capable of taking up fungal antigen, CD11c-enriched cells from BM and spleen were co-cultured for 2 h with FITC-labelled zymosan, a glycan derived from the cell wall of *Saccharomyces cerevisiae*, and uptake of zymosan by DCs was determined by flow cytometry [28] (Figure 3C); Imagestream analysis showed that the zymosan was inside the cells, indicating that both BM DC and splenic DC can phagocytose the zymosan (Figure S4). We discovered that uptake of zymosan by BM DCs was dose-dependent and significantly higher than uptake of zymosan by splenic DCs (Figure 3D), culminating in ~35% zymosan^+^ BM DCs compared to ~7% zymosan^+^ splenic DCs. The vast majority of zymosan uptake by BM DCs was mediated through Dectin-1, as zymosan^+^ BM DCs were reduced to ~5% when the co-culture was performed in the presence of a Dectin-1 blocking antibody (Figure 3E). Thus, BM DCs express high, functional protein levels of the c-type lectin receptor Dectin-1. To determine whether Dectin-1 on BM DCs also mediates uptake of live fungi, we co-cultured CD11c-enriched cells from BM and spleen overnight with live, FITC-labeled *Aspergillus conidia* in the presence or absence of a Dectin-1 blocking antibody. We found that *Aspergillus* was taken up by ~25% of BM DCs and ~10% of splenic DCs, and uptake was reduced to ~5% in DCs in both organs when Dectin-1 was blocked (Figure 3F). Together, these data indicate that, in vitro, murine BM DCs are particularly capable of taking up both inactive fungal antigens and live fungi through Dectin-1.

### 3.5. DCs in Human BM Also Take Up Zymosan, Which Is CR3-Mediated

**Figure 4:**
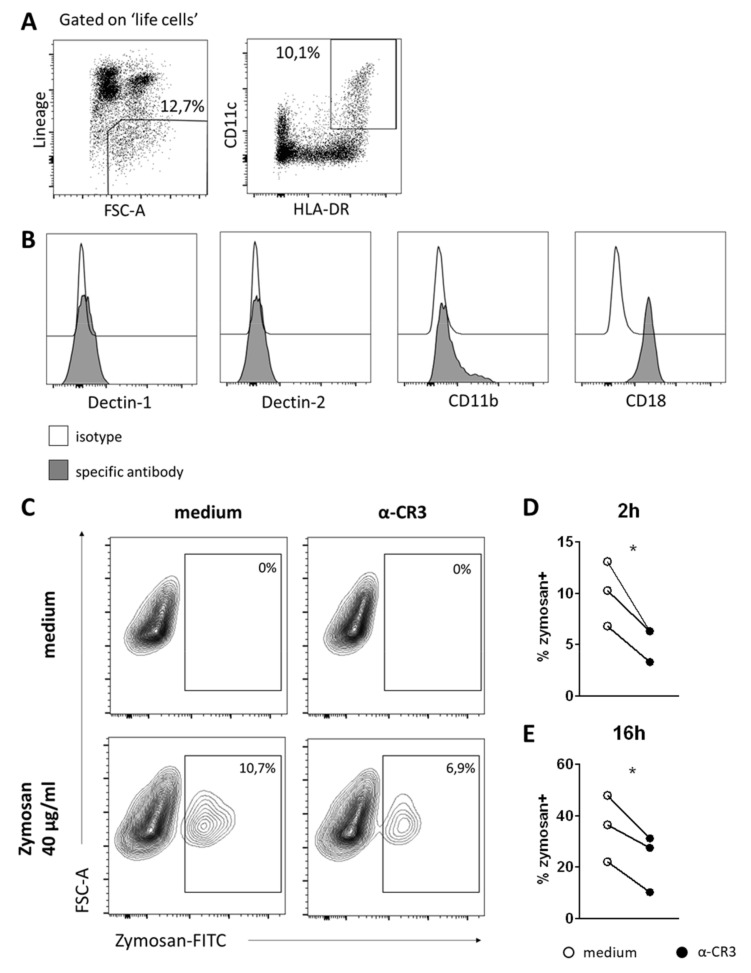
Human BM DCs take up zymosan in a CR3-dependent manner. (**A**) Representative FACS plots of DCs in human BM aspirates. (Lineage: CD3, CD19, CD20, CD14, CD16, CD56). (**B**) Representative histograms of geoMFI for expression of Dectin-1, Dectin-2, CD11b, and CD18 expression by human BM DCs. Representative FACS plots (**C**) and quantification of uptake of zymosan by human BM DCs after 2 h (**D**) and overnight (**E**) in vitro co-culture in the presence or absence of CR3 blocking antibodies (Mean ± SD, *n* = 3, paired *t*-test). * *p* < 0.05.

To correlate our findings to the human situation, we examined whether human BM also harbors resident DCs that can take up fungal antigens. We could identify a substantial population of myeloid dendritic cells in human BM aspirates (Figure 4A). These human BM DCs did not express detectable levels of either Dectin-1 or Dectin-2, but instead expressed the β2 integrin CD11b/CD18 or CR3 (Figure 4B), which is the most important receptor for the recognition of β-glucan in humans [17]. Co-culture of CD11c-enriched mononuclear cells from human BM aspirates with zymosan revealed that human BM DCs were particularly capable of taking up zymosan in a CR-3 dependent manner (Figure 4C–E). Thus, both murine and human BM DCs are capable of taking up fungal antigens through β-glucan-specific receptors.

### 3.6. Zymosan Is Taken Up by BM DCs after In Vivo Administration and Boosts the Number of Neutrophil Progenitors in BM

**Figure 5:**
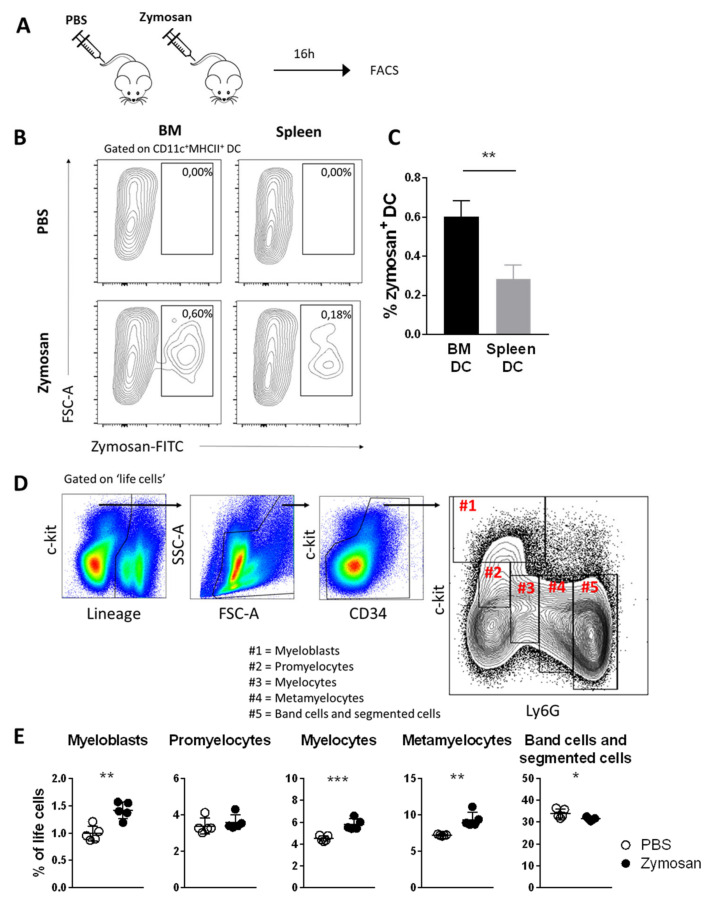
Zymosan is taken up by BM DCs after in vivo administration and boosts the number of neutrophil progenitors. (**A**) Experimental set-up, (**B**) representative FACS plots, and (**C**) quantification of in vivo uptake of zymosan particles by BM DCs and splenic DCs, 16 h after intravenous administration of zymosan. (Mean ± SD, *n* = 5, paired *t*-test). (**D**) Gating strategy for neutrophil precursors. (**E**) Quantification of neutrophil precursors in murine BM, 16 h after intravenous administration of zymosan. (Mean ± SD, *n* = 5 per group, unpaired *t*-test). * *p* < 0.05; ** *p* < 0.01; *** *p* < 0.001.

We next injected mice intravenously with FITC-labeled zymosan and analyzed DC from spleen and BM 16 h after injection. Similar to the results of our in vitro experiments, zymosan uptake in vivo by BM DCs was significantly higher than zymosan uptake by splenic DCs (Figure 5A–C). However, uptake of zymosan in vivo was much lower than what we observed in vitro, possibly because of low bioavailability of zymosan in the BM. We found that, in total, 0.22–0.28 % of nucleated cells in the BM had taken up zymosan (Figure S4A,B). In addition, we could identify a population of free, non-phagocytosed zymosan that constituted 0.24–0.39% of total events, indicating that one third to half of all the zymosan molecules that reached the BM were taken up by BM cells (Figure S5C,D). Of all the BM cells that had phagocytosed zymosan, ~10% were neutrophils and ~7% were DCs (Figure S5A,B). Thus, BM DCs are capable of taking up zymosan in vivo and in vitro.

Neutrophils are the most important immune cells in the defense against fungal infection, and many studies have reported neutrophilia and emergency granulopoiesis upon exposure to fungal antigens [29,30,31]. Accordingly, we found that in vivo administration of zymosan induced expansion of granulocyte-precursors in the BM. We could discern 5 distinct subpopulations of granulocyte precursor-cells based on a previously reported gating strategy, specifically myeloblasts, promyelocytes, myelocytes, metamyelocytes, and band- and segmented cells (Figure 5D) [29]. Sixteen hours after intravenous administration of zymosan, we observed an increase in early granulocyte precursors (myeloblasts, myelocytes, and metamyelocytes) in the BM, whereas the levels of more mature band cells and segmented cells were slightly decreased (Figure 5E). Interestingly, we found no signs of extramedullary hematopoiesis in the spleen (data not shown), but could also not observe an increase in the number of mature neutrophils in the spleen on either day 1 or day 3 (Figure S5E). These data suggest that intravenous administration of zymosan modestly enhances granulopoiesis in the bone marrow, but that this is not sufficient to have a strong impact on the number of circulating neutrophils.

### 3.7. BM DCs Promote Neutrophil Formation in a G-CSF Dependent Manner upon Exposure to Zymosan

**Figure 6:**
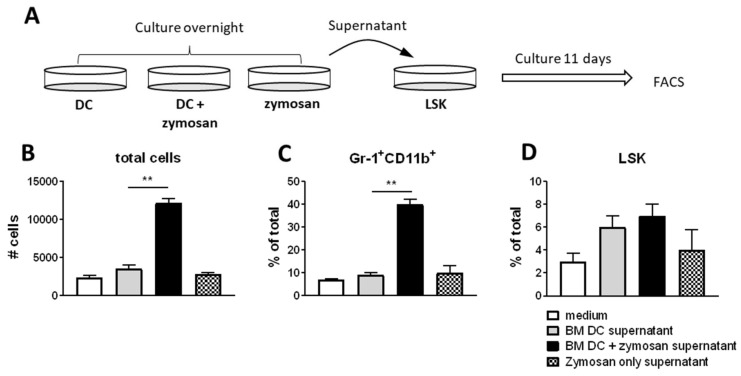
BM DCs promote neutrophil formation in a G-CSF dependent manner upon exposure to zymosan. (**A**) Experimental set-up of in vitro LSK cell cultures (**B**) Total number of cells, (**C**) percentage of LSK, and (**D**) percentage of CD11b^+^GR-1^+^ neutrophil type cells after 11 days in vitro culture of LSK cells with supernatant of BM DCs and zymosan cocultures. Graphs depict mean ± SD of experimental duplicates and are representative of 4 independent experiments. (unpaired *t*-test). Gating strategy for LSK cells and neutrophil type cells after culture can be found in Supplementary Figure S6. Graphs depict mean ± SD of experimental duplicates and are representative of three independent experiments. (unpaired *t*-test). ** *p* < 0.01.

To investigate whether and how zymosan-stimulated BM-resident DCs can contribute to neutrophil formation, we co- cultured sorted BM DCs overnight with zymosan, and harvested the supernatant (Figure 6A). This supernatant was subsequently added to a long-term in vitro culture of sorted Lineage^−^c-kit^+^sca-1^+^ (LSK) cells in the presence of hematopoietic supporting cytokines SCF, TPO and FLT3L. After 11 days of culture, the total number of cells that could be retrieved was significantly higher when LSK were cultured with supernatant from zymosan-stimulated BM, as compared to LSK cells that were cultured with supernatant from BM DCs only (Figure 6B). Importantly, this enhanced expansion was not due to residual fungal antigen in the supernatant, as supernatant from zymosan only did not enhance hematopoiesis (Figure 6B). Supernatant from zymosan-stimulated BM DCs also increased the percentage of Gr-1^+^CD11b^+^ neutrophil-type cells in the culture from 10% to 40% (Figure 6C). These cells had high expression of GR-1, and lacked expression of F4/80, indicating that they were indeed neutrophilic granulocytes, rather than monocytes, macrophages or eosinophils (data not shown). The increase of neutrophil-type cells was not accompanied by LSK cell exhaustion, as LSK cells still constituted 4–6% of the total cell culture after 11 days irrespective of the culture condition (Figure 6D). Thus, it can be concluded that zymosan-stimulated BM DCs produce soluble factors that skew the hematopoietic process towards neutrophil formation without inducing LSK cell exhaustion.

Finally, we set out to determine the identity of the molecule(s) produced by zymosan-stimulated BM DCs that induced increased granulopoiesis by LSK cells. The obvious candidate was granulocyte-colony-stimulating-factor (G-CSF), which is pivotal for homeostatic and emergency granulopoiesis [32]. We therefore added a G-CSF blocking antibody to LSK cultures with supernatant from BM DCs cultured with and without zymosan. Blocking G-CSF function reversed the increase in total hematopoietic output of LSK cultures with supernatant from zymosan-stimulated BM DCs (Figure S6C). This translated into a significant reduction of the percentage of neutrophil-type cells in the culture (Figure S6D), whereas the percentage of LSK cells remained the same (Figure S6E). As expected, we observed no significant effect of the G-CSF blocking antibody in LSK cultures with supernatant of BM DCs cultured without zymosan (Figure S6C–E). Thus, BM DCs that are exposed to fungal antigens can skew the hematopoietic process towards increased neutrophil formation in a G-CSF dependent manner. The levels of G-CSF that are produced by zymosan-stimulated BM DCs and that are needed to increase neutrophil output are most likely very low, as we could not detect G-CSF in the supernatant of overnight BM DC co-cultures with zymosan (ELISA with detection limit 4 pg/mL; data not shown). Together, our data reveal an as yet unappreciated role for BM DCs as translators between fungal infection and the hematopoietic process.

## 4. Discussion

Our findings that the BM contains a unique population of resident DCs are in agreement with recent data by the Link lab, who also showed that most DCs in BM are perivascularly located (~50% of the cells are located <10 μm from the nearest sinusoid) and largely have a mature cDC2 phenotype [33]. They also demonstrated that depletion of DCs induces the mobilization of HSPCs from the BM by acting on the integrity of and chemokine expression by the vascular niches [33]. These findings also suggest a crosstalk between DCs and HSPCs, which is further supported by the fact that treatment of mice with the TLR7/8 agonist R848 directly affects the behavior and function of HSPCs and induces an expansion of the number of BM-resident DCs [34]. Our findings in this study take this crosstalk between HSPCs and DCs one step further, as we show that BM-resident DCs are particularly capable of taking up fungal antigens and subsequently skew hematopoiesis towards increased production of neutrophils in a G-CSF dependent manner. We initially hypothesized that BM DCs might not respond strongly to inflammation or infection, in order to minimize immune activation in the BM. However, we found that BM DCs in fact respond effectively to systemic inflammatory agents and that they have an inflammatory transcriptional profile, with particularly high expression of c-type lectin receptors. We discovered that BM DCs were efficient in taking up zymosan molecules through Dectin-1 in mice and CR3 in humans; although human BM DCs use CR3 rather than Decin-1, it could be that CR3 is also involved in murine BM DCs, as this is highly expressed on these cells. In fact, Dectin-1 has been shown to activate CR3 on murine neutrophils [35], but we did not examine the role of CR3 on murine BM DCs. We did find that supernatant of zymosan-stimulated BM DCs enhanced neutrophil formation in LSK cultures in a G-CSF dependent manner. It remains to be investigated how these findings translate to the in vivo situation; we found that a single intravenous injection of zymosan is sufficient to enhance granulopoiesis in the BM, but this did not increase the number of circulating neutrophils in the spleen. We have not tested whether zymosan injection can increase the number of circulating neutrophils in peripheral blood, but based on the spleen data it could be anticipated that a single injection of zymosan is not sufficient for this to occur. It would though be interesting to examine whether repeated injections with zymosan (or *Aspergillus conidia*) can induce this and elicit the development of emergency neutropoiesis. So, although our findings may be a first indication that resident BM DCs can act as translators between infection and the hematopoietic process, this hypothesis is highly speculative, as it still remains to be shown whether BM DCs are required and/or sufficient to boost neutrophil formation, in particular during a real fungal infection.

While it is undisputed that G-CSF is required for normal homeostatic and emergency granulopoiesis [32], the physiological source of G-CSF in the BM remains uncertain. Many cell types, including stromal cells, endothelial cells, and macrophages, have the ability to express G-CSF, but their production of the G-CSF protein is highly regulated and not constitutive [36]. As we found that BM DCs can skew hematopoiesis towards production of neutrophils upon exposure of zymosan, we postulate that BM DCs can mediate G-CSF driven feedback to the hematopoietic system to increase neutrophil production when the situation demands it. Boettcher et al. showed that BM endothelial cells produce high levels G-CSF upon TLR4 triggering by LPS or *E. coli*, which induced HSPC skewing and accelerated granulopoiesis [37]. DCs may be one of the cellular sources of G-CSF in the BM that can enhance neutrophil formation upon infection, and the source may vary depending on the type of pathogen. Alternatively, BM DC may indirectly induce G-CSF signaling in hematopoietic progenitors. In this scenario, zymosan-stimulated BM DC produce other cytokines or soluble factors that subsequently induce the production of autocrine-signaling G-CSF by hematopoietic progenitor cells. This is compatible with the results from our in vitro LSK cultures and would explain why we could not detect G-CSF in the supernatant of zymosan-stimulated DC cultures. Alternative explanations could be that low levels of G-CSF (below the detection limit of the ELISA) have an effect, in particular if this is in synergy with other secreted growth factors, or that the secreted G-CSF is bound by a receptor, which blocks detection by the ELISA, while maintaining its functional effect on the progenitor cells. A final possibility is that GM-CSF, rather than G-CSF, is induced by zymosan, and that the anti-G-CSF antibody is cross-reactive to GM-CSF, thus causing the observed effect. Clearly, this remains speculative and more conclusive experiments are necessary to investigate the molecular mechanism by which BM DCs enhance neutrophil formation.

Stark et al. have previously reported an IL-23 dependent role for DCs in normal homeostasis of neutrophils [38]. They found that BM-derived DCs produce high levels of IL-23 when they take up apoptotic neutrophils, and thereby induce the production of IL-17 by T cells, which is key in the maintenance of normal neutrophil homeostasis. The authors suggested that the increase in IL-17 induced G-CSF production by other, undetermined cells, which would increase granulopoiesis to balance neutrophil loss through apoptosis. Jiao et al. also reported a role for DCs in neutrophil homeostasis, as they found that systemic depletion of dendritic cells in CD11c-DTR mice results in increased levels of peripheral neutrophils [39]. They concluded that loss of DCs results in increased BM mobilization and peripheral recruitment of neutrophils, as they found that the BM of DC-depleted mice contained only few neutrophils. While these studies focused on different aspects of DC-neutrophil interaction, they do support the notion that DCs play an as of yet underappreciated role in the regulation of neutrophil homeostasis.

The results of our study may also be of importance for the concept of “trained immunity”. While the innate immune system has classically been viewed as a non-specific first line of defense that lacks the ability to form immunological memory, it is now more and more accepted that innate immune cells can mount resistance to reinfection through genomic imprinting and reprogramming [40]. Recent studies have shown that, in addition to functional changes of mature innate cells in the periphery, the formation of innate memory can be inflicted through modulation of HSPC differentiation [41,42]. Mitroulis et al. showed that administration of β-glucan induces increased myelopoiesis, which results in a beneficial response to secondary systemic inflammation [42]. However, although HSCs express a variety of PRRs through which they can sense infectious agents [43], they do not express Dectin-1 [44]. The effect of β-glucan on hematopoiesis is therefore likely to be indirect, and indeed Mitroulis et al. found it to be associated with IL-1β and GM-CSF (granulocyte-macrophage colony-stimulating factor) signaling. Although they also observed increased G-CSF levels in the BM of mice that were exposed to β-glucan, they did not investigate whether G-CSF was involved in the durable modulation of HSPC differentiation [42]. Yet, it is conceivable that G-CSF produced or induced by BM DCs upon fungal infection inflicts a lasting increase in HSPC differentiation towards the myeloid and the granulocyte lineage, thereby creating a cellular environment that it resilient to fungal reinfection. It would therefore be interesting for future studies to investigate the effect of fungal antigen-loaded BM DCs on the epigenetic profile of HSPC, and to determine whether these HSPC continue to produce increased numbers of neutrophils for longer periods of time.

Finally, our findings are relevant in the context of clinical hematopoietic stem cell transplantation (HSCT). Opportunistic fungal infections are a common complication after HSCT, especially during the early neutropenic phase and severe graft-versus-host disease, and are a major cause of morbidity and mortality [45]. These opportunistic infections are largely caused by the lack of sufficient peripheral neutrophils, and the results of our study imply that slow DC recovery after HSCT [46] might be part of the problem. While G-CSF is commonly used after autologous HSCT and occasionally after allogeneic HSCT to minimize prolonged neutropenia and its associated morbidity and mortality [47,48,49], administration of G-CSF is not without risk [49,50]. It would therefore be interesting to investigate whether expansion of the DC compartment through FLT3L administration after HSCT [51] can restore successful regulation of emergency granulopoiesis in neutropenic patients.

## 5. Conclusions

We have shown that resident BM DCs function not only as initiators of adaptive immune responses, but also as translators of infectious and inflammatory signals to skew hematopoiesis. These data contribute to our understanding of the concept of trained immunity and provide a new angle for the increased susceptibility for fungal infections under conditions of BM damage. We have only begun to unravel the relationship between BM DCs and the hematopoietic process, and we have hereby opened up a new avenue of research for future studies that will be of biological and clinical importance.

## Data Availability

The RNA sequencing data presented in this study will be made available at GEO; accession numbers will be provided during review.

## Supplementary Materials

The following are available online at https://www.mdpi.com/article/10.3390/cells11010055/s1, Figure S1: DCs in BM and spleen, Figure S2: CD8 T cell activation by BM DCs, Figure S3: Gene ontology enrichment analysis of RNAseq from BM DCs vs splenic DCs, Figure S4: Imagestream analysis of zymosan uptake by DC, Figure S5: In vivo zymosan uptake by BM cells & impact on neutrophil numbers, Figure S6: Gating strategy for LSK cultures and impact of blocking G-CSF, Table S1: Genes differentially expressed between DCs in BM vs. spleen.
