# Supplementary material for: Bone Marrow Harbors a Unique Population of Dendritic Cells with the Potential to Boost Neutrophil Formation upon Exposure to Fungal Antigen
Source: Cells. 2021 Dec 24;11(1):55. doi: 10.3390/cells11010055 (PMC8750392; doi:10.3390/cells11010055)
Supplement: Supplementary file 1 [file cells-11-00055-s001.zip › BM DC Manuscript for Cells Suppl figures vs3.pdf]

Supplemental Figures S1-S6, belonging to manuscript:

Bone marrow harbors a unique population of dendritic cells that promotes granulopoiesis upon exposure to fungal antigens.

by Marieke Goedhart *et al.*

Supplemental figure 1

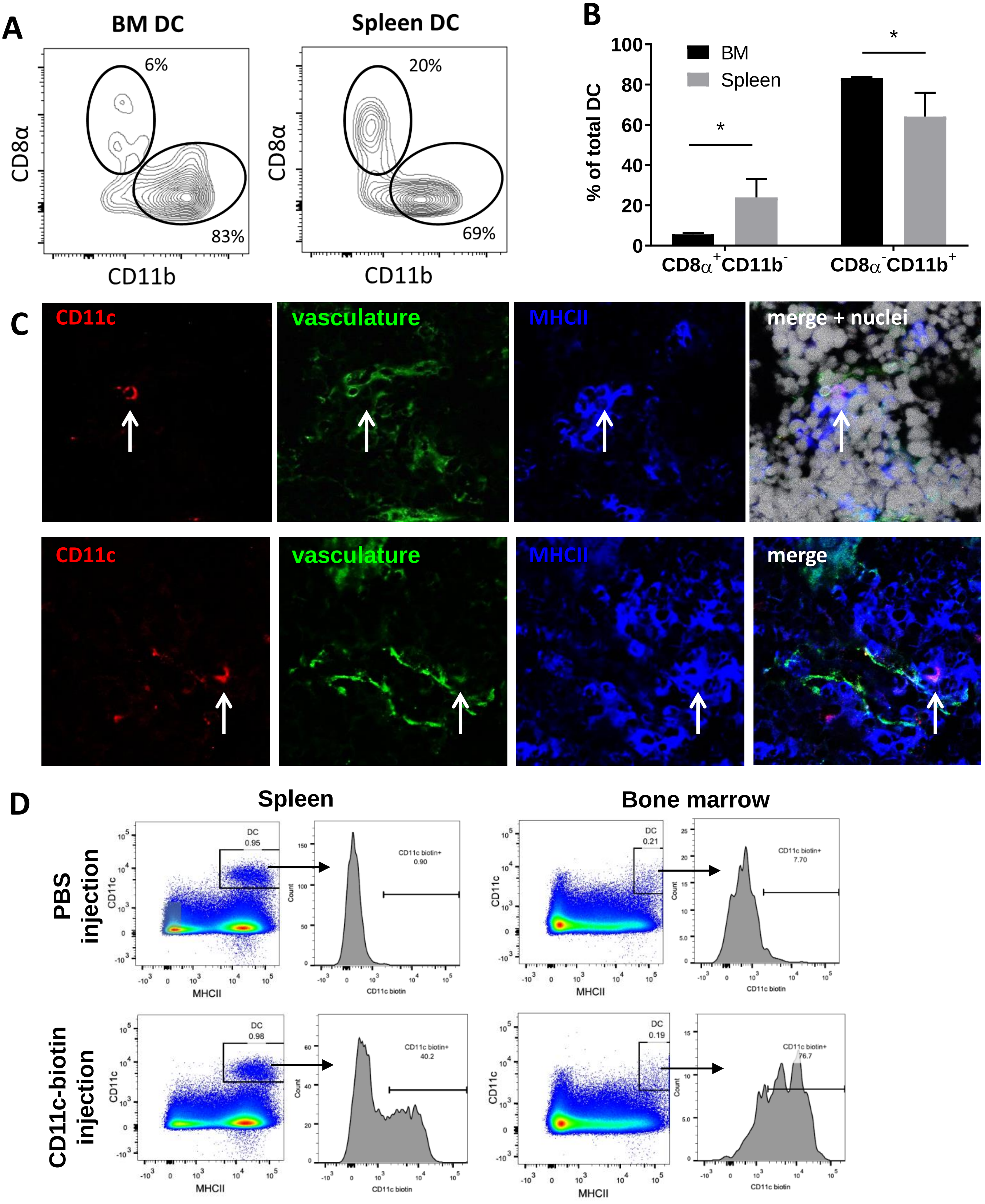

Figure S1. DCs in BM and spleen

(A) Representative FACS plots and (B) percentages of DC subsets based on CD8 and CD11b expression in BM and spleen; (Mean ± SD, n=3, paired t-tests). *P < 0.05; **P < 0.01; ***P < 0.001. Gating strategy for panel A: Dead cells and doublets were excluded by FSC-SSC gating, followed by selecting for $CD11c^{hi}MHCII^{+}$ cells as the DC population. (C) More representative images of immunofluorescent imaging for DCs in BM (following Figure 1E); CD11c (red), VE-cadherin and CD31 (green), MHCII (blue) and counter-stained with Hoechst to visualize nuclei (grey; only in top row). (D) Gating strategy of Figure 1G.

Supplemental figure 2

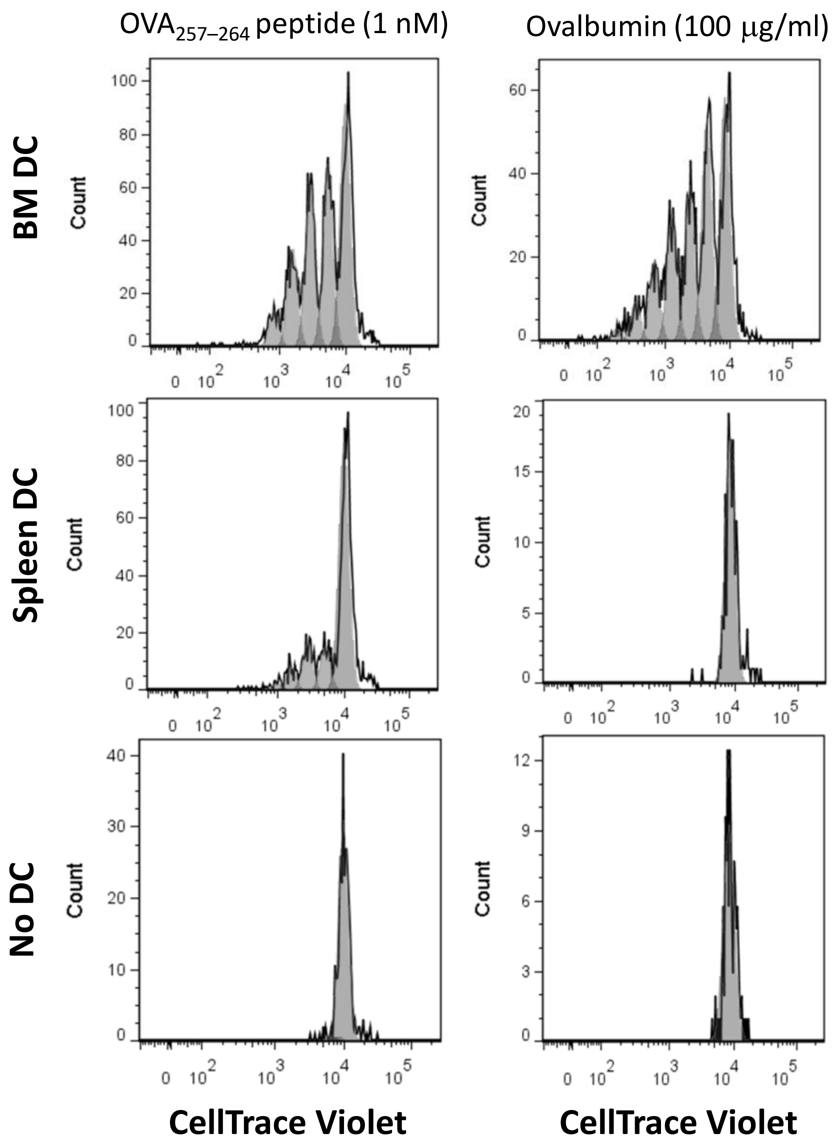

Figure S2. CD8 T cell activation by BM DCs

CellTrace Violet labelled naïve OT-I CD8 T cells were stimulated with BM DC (top), spleen DC (middle) or no DC (bottom), that were loaded with either $OVA_{257–264}$ peptide (1nM) or Ovalbumin protein (100 μg/ml). Initial ratio of T cells: DC at day 0 was 2:1. Dilution of CellTrace Violet in the T cells was measured after 3 days of culture. FACS plots are representative of experimental triplicates.

Supplemental figure 3

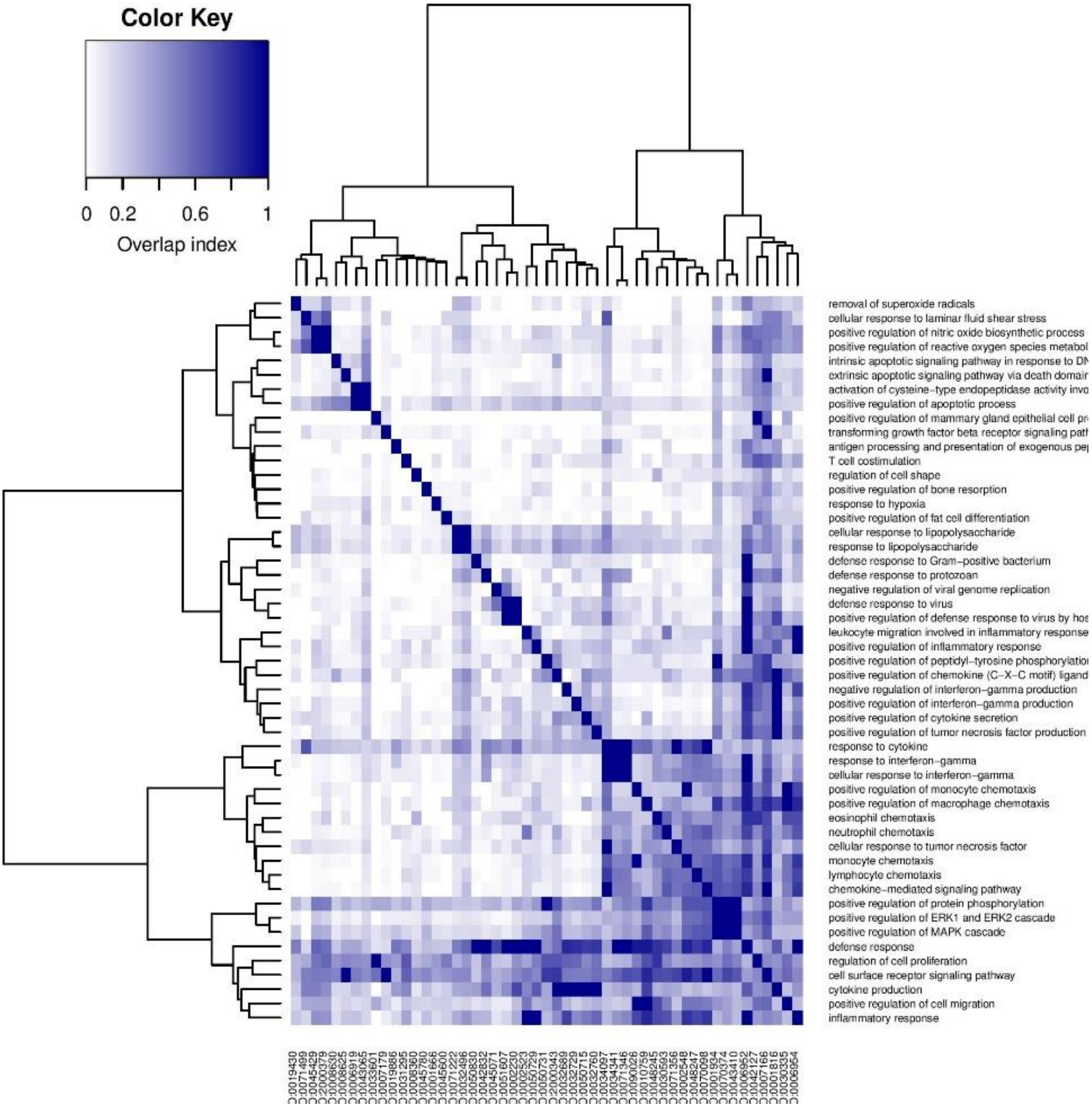

Figure S3. Gene ontology enrichment analysis of RNAseq from BM DCs vs splenic DCs
Hierarchical clustering of all significantly enriched GO-terms shown in a heatmap.

Supplemental figure 4

BM

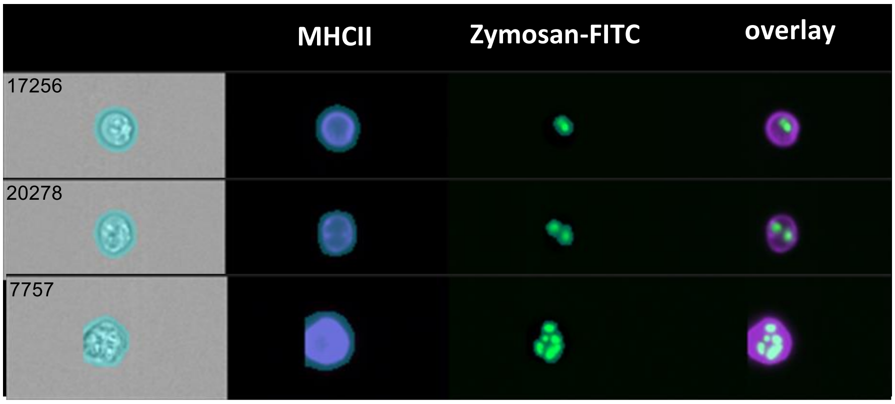

Spleen

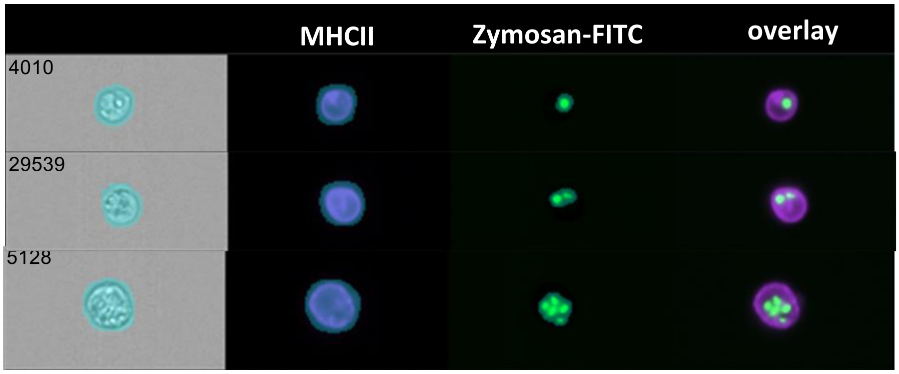

Figure S4. Imagestream analysis of zymosan uptake by DC
Representative images from ImageStream analysis of BM DCs and splenic DCs that were co-cultured with zymosan for 2 hours.

Supplemental figure 5

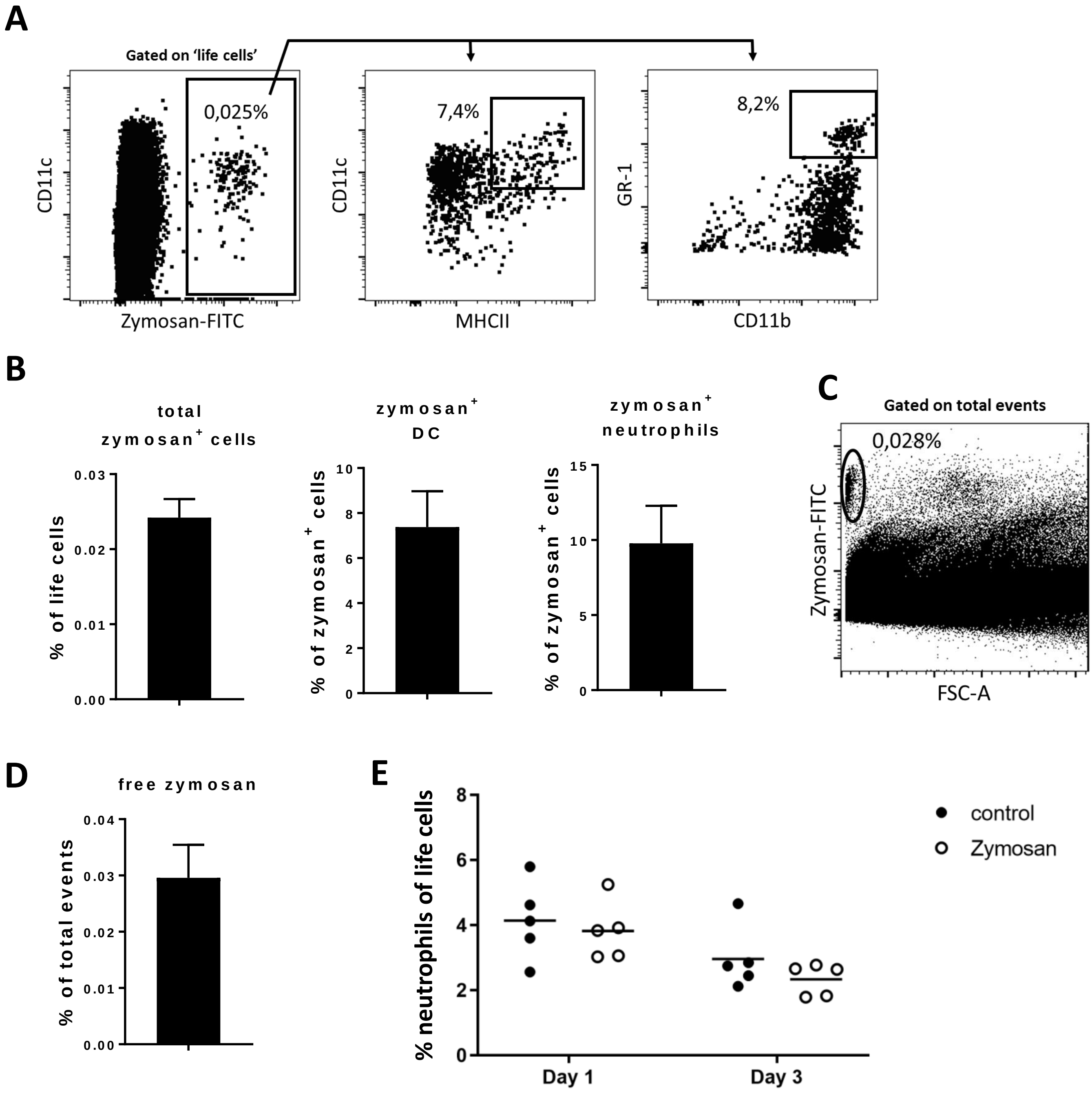

Figure S5. ***In vivo*** **zymosan uptake by BM cells & impact on neutrophil numbers**
(A) Gating strategy for (B) quantification of zymosan^{+} BM cells, 16 hours after intravenous administration of zymosan. (C) Gating strategy for (D) quantification of free, not phagocytosed zymosan particles in BM, 16 hours after intravenous administration of zymosan. (E) Quantification of the number of mature neutrophils in the spleen after 16 hours (1 day) or 3 days.

Supplemental figure 6

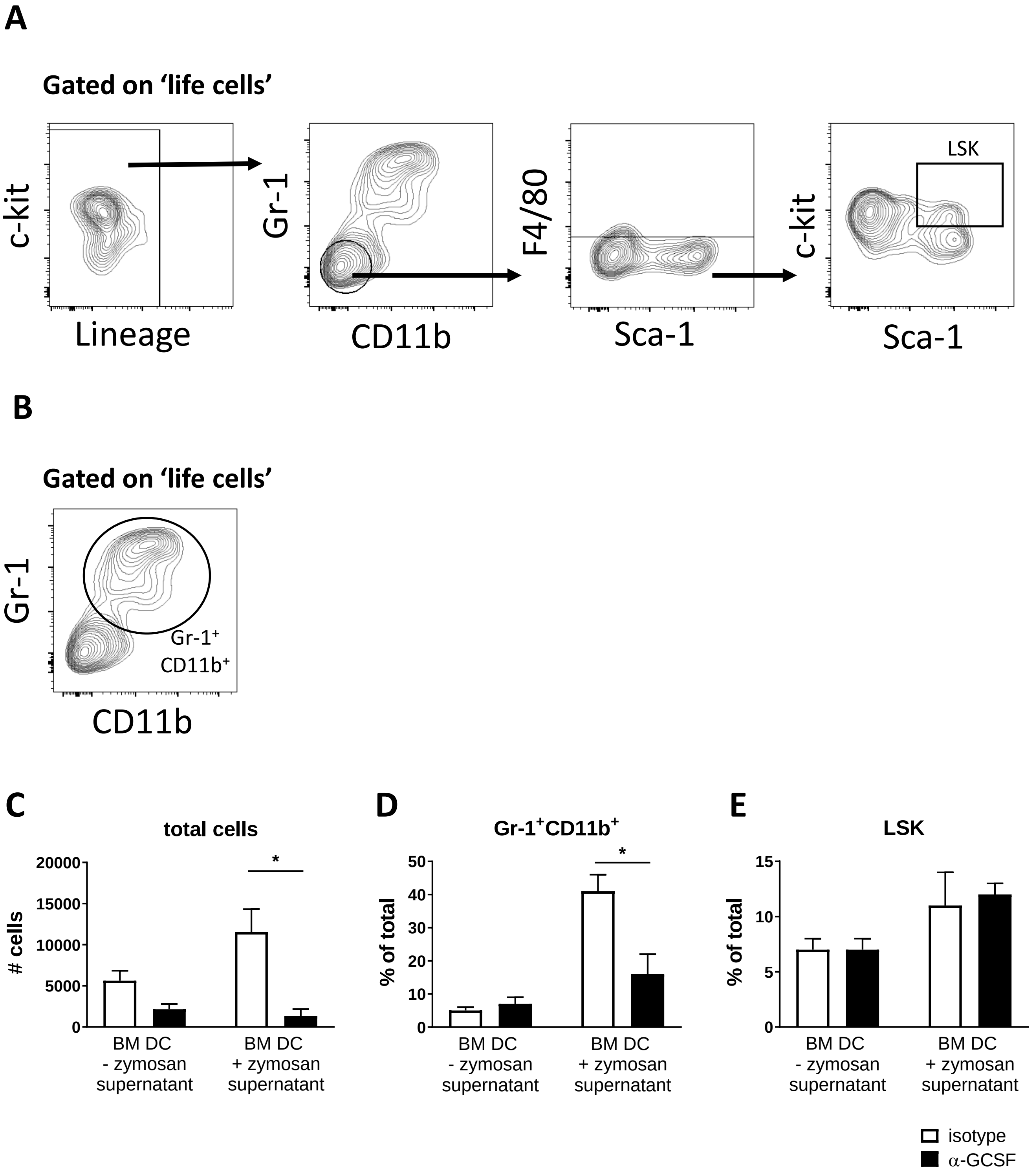

Figure S6. Gating strategy for LSK cultures and impact of blocking G-CSF. Gating strategy for (A) LSK cells and (B) Gr-1+CD11b+ neutrophil type cells after 11 days *in vitro* culture of LSK cells in the presence of BM DC supernatant. Lineage: CD4, CD8, B220, Ter119, Gr-1, CD11b. (C) Total number of cells, (D) percentage of LSK and (E) percentage of CD11b+GR-1+ neutrophil type cells after 11 days in vitro culture of LSK cells with supernatant of zymosan-stimulated BM DCs, in the presence of a G-CSF blocking antibody or isotype control. Graphs depict mean ± SD of experimental duplicates and are representative of 3 independent experiments. (Unpaired t-test). *P < 0.05; **P < 0.01; ***P < 0.001
